# Impact of COVID-19 on the Microbiome and Inflammatory Status of Type 2 Diabetes Patients

**DOI:** 10.3390/biomedicines11010179

**Published:** 2023-01-11

**Authors:** Gratiela Gradisteanu Pircalabioru, Georgiana Alexandra Grigore, Ilda Czobor Barbu, Mariana-Carmen Chifiriuc, Octavian Savu

**Affiliations:** 1 Research Institute of University of Bucharest (ICUB), 050095 Bucharest, Romania; 2 Academy of Romanian Scientists, 050045 Bucharest, Romania; 3 Faculty of Biology, University of Bucharest, 050095 Bucharesti, Romania; 4 Romanian Academy, 010071 Bucharest, Romania; 5 “N.C. Paulescu” National Institute of Diabetes, Nutrition and Metabolic Diseases, 020042 Bucharest, Romania; 6 Department of Doctoral School, “Carol Davila” University of Medicine and Pharmacy, 5th District, 050474 Bucharest, Romania

**Keywords:** microbiome, microbiota, mycobiome, COVID-19, type 2 diabetes

## Abstract

The severe acute respiratory syndrome–related coronavirus 2 (SARS-CoV-2) pandemic has advanced our understanding of the host–microbiome–virus interplay. Several studies in various geographical regions report that SARS-CoV-2 infection disrupts the intestinal microbiota, allowing pathogenic bacteria such as *Enterobacteriaceae* to thrive, and triggering more severe disease outcomes. Here, we profile the microbiota of 30 individuals, 15 healthy controls and 15 type 2 diabetes (T2D) patients, before and after coronavirus disease 2019 (COVID-19). Despite similar viral loads in both patients and controls, SARS-CoV-2 infection led to exacerbated microbiome changes in T2D patients, characterized by higher levels of *Enterobacteriaceae*, loss of butyrate producers and an enrichment in fungi such as *Candida* spp. and *Aspergillus* spp. Several members of the microbiota were associated with more severe clinical and inflammatory (IL-8 and IL-17) parameters. Future studies to delineate the connection between cytokine release and microbiota disturbances will enhance our understanding of whether these microbial shifts directly impact the cytokine storm in COVID-19 patients or whether they are consecutive to the critical disease.

## 1. Introduction

Deciphering the pathological mechanisms underlying SARS-CoV-2 infection is an urgent global priority, especially in high-risk patients, including type 2 diabetes (T2D) patients. SARS-CoV-2 infects the lungs after binding on the ACE2 receptors from the alveolar epithelial cells, causing pneumonia that will eventually progress to acute respiratory distress syndrome (ARDS), particularly in elderly and other categories of immune-compromised patients [1]. Severe coronavirus disease 2019 (COVID-19) was reported to be associated with an exacerbated immune response, which may trigger systemic organ failure [2]. 

The microbiota has been reported to impact pulmonary health through the bidirectional communication between the gut microbiota and the lungs, often referred to as the “gut-lung axis” [3]. Interestingly, SARS-CoV-2 RNA was found in the feces of infected patients, suggesting a subtle link between the lung and the intestine [4,5,6]. Indeed, changes in gut microbiome signatures are reported for COVID-19 patients, particularly in patients treated with antibiotics during hospitalization [6,7].

Even in mild infections, gastrointestinal symptoms are frequently reported. COVID-19 patients exhibit a loss of commensal microbes during hospitalization [8,9,10], and persistent microbiota changes were described in patients with long-term complications from COVID-19 [11,12]. 

These studies suggest that microbiota diversity and the presence of beneficial microbes in the gut may harbor an important role in determining the course of COVID-19. 

Taking into account that patients with co-morbidities such as cardiovascular disorders and T2D are less efficient in fighting SARS-CoV-2 infection [13,14], the aim of this study was to investigate the microbiome alterations triggered by COVID-19 in a T2D cohort compared to healthy controls. 

## 2. Materials and Methods

### 2.1. Study Group

The study population was represented by 30 individuals (15 T2D patients from the National Institute of Diabetes, Nutrition and Metabolic diseases N.C. Paulescu from Bucharest, Romania and 15 healthy volunteers). The exclusion criteria for the controls were the use of antibiotics, laxatives or antidiarrheal drugs in the past 6 months, and known complex infections, sepsis, malignant disease, AIDS, pregnancy and bowel surgery in the past 6 months. All participants included in the study signed an informed consent. Fecal samples were collected between 3 to 7 days from the COVID-19 diagnostic. No cases of COVID-19 reinfection were reported for the analyzed cohort. The study was conducted according to the guidelines of the Declaration of Helsinki and approved by the Ethics Committee of University of Bucharest (protocol code CEC reg. no 235/9.10.2019). The characteristics of the subjects included in the study are listed in Table 1.

### 2.2. Microbiota Analysis

Stool samples were collected in the morning using antiseptic handling and immediately frozen at −20 °C. DNA was extracted using the DNA Stool Mini kit (Qiagen) following the manufacturer’s instructions. For microbiome sequencing, partial 16S rRNA gene sequences were amplified using primer pairs targeting the hypervariable regions of the 16S rRNA gene (V2-4-8 and V3-6, 7-9). The obtained amplicons were further purified using Agencourt AMPure beads (Beckman coulter, Brea, CA, USA) and a DynaMag magnet. Libraries were generated using the Ion Plus Fragment Library kit (Applied Biosystems) and quantified using the Taqman Ion Universal Library Quantitation kit (Cat no. A26217). Sequencing template preparation was conducted using the Ion PGM Hi-Q View OT2 kit-400. Sequencing of the amplicon libraries was performed on a 316-chip using the Ion Torrent PGM system. Next, the obtained individual sequence reads were filtered by the Ion Reporter PGM software to discard polyclonal and low-quality reads. The sequencing data were processed using the Quantitative Insights Into Microbial Ecology (QIIME) [15] pipeline. 16S rRNA Operational Taxonomic Units (OTUs) were defined at ≥97% sequence homology. All reads were classified using reference datasets (Curated Greengenes v13.5; Curated MicroSEQ(R) 16S Reference Library v2013.1). The alpha and beta diversity graphics created in QIIME were exported from the Ion Reporter Software. For alpha diversity, the Shannon curves were generated to analyze species diversity within the samples.

### 2.3. Real Time PCR

COVID-19 diagnostic was performed using the Genesig^®^ COVID-19 2G (PRIMER DESIGN, Eastleigh, UK) kit from nasopharyngreal swabs. Human peripheral blood mononuclear cells (PBMCs) were isolated using Ficoll gradient centrifugation from blood samples collected from SARS-CoV-2 infected patients. PBMCs were further used for RNA isolation using a commercial kit (PureLink RNA Mini Kit, Invitrogen, Waltham, MA, USA). The RNA obtained was further reverse transcribed using the High-Capacity Reverse Transcription kit (Applied Biosystems, Waltham, MA, USA). Real time PCR for Nox1, Nox2, Nox4, IL-17 was performed using commercially available Taqman probes (Applied Biosystems)—Hs00166163_m1, Hs01071086_g1, Hs04980925_m1, Hs02786624_g1. GAPDH was used as internal control. Detection of fungal population abundance was performed using SYBR Green (Applied Biosystems) and the specific primers listed in Table 2. Fungal rRNA 18S primers were used as an internal control.

### 2.4. ELISA 

Cytokine detection was performed on serum samples using commercially available kits: Human Interleukin-1beta (Hu IL-1beta) ELISA kit (Thermo Scientific, Cat. No. KHC0011), Human IL-8 ELISA kit (Cat. No. KHC0081), C Reactive Protein (CRP) kit (Cat. No. KHA0031). Detection of butyrate was performed using a commercially available kit (Sigma Aldrich, St. Louis, MO, USA)

### 2.5. Reactive Oxygen Species (ROS) and Nitric Oxide Synthase (NOS) Detection 

Detection of reactive oxygen species (hydrogen peroxide) was performed on isolated PBMCs using Amplex Red (Thermo Scientific, Waltham, MA, USA). PMA (Phorbol 12-myristate 13-acetate) was used as a stimulus for ROS production. Detection of NOS was performed on serum samples using a kit based on the Griess reaction (Nitric oxide kit, Cat no. EMSNO, Invitrogen).

### 2.6. Statistical Analysis

Data are shown as mean ± SEM and were graphed using GraphPad Prism 9.4.1. Differences in viral RNA, ROS and cytokine levels were tested using Unpaired *t*-test with Welch’s correction. Statistical differences in gene expression and microbe abundance and diversity in uninfected and infected individuals were quantified using 1-way ANOVA post hoc Bonferroni test. A heat map based on Spearman correlations was constructed to compare the microbiota patterns and clinical parameters. The *p* values *<* 0.05 were considered as statistically significant. The statistical significance levels were *, *p* < 0.05; **, *p* < 0.01; ***, *p* < 0.001, **** *p* < 0.0001

## 3. Results

In 2019, before the SARS-CoV-2 pandemic, we initiated a national study to analyze the microbiome patterns in T2D. In this purpose, we collected stool samples from a cohort of 105 T2D patients and 45 controls individuals. During the COVID-19 pandemic, we re-evaluated the microbiota composition for a subset of patients who tested positive for COVID-19 and gave their consent to participate in our study.

**Figure 1:**
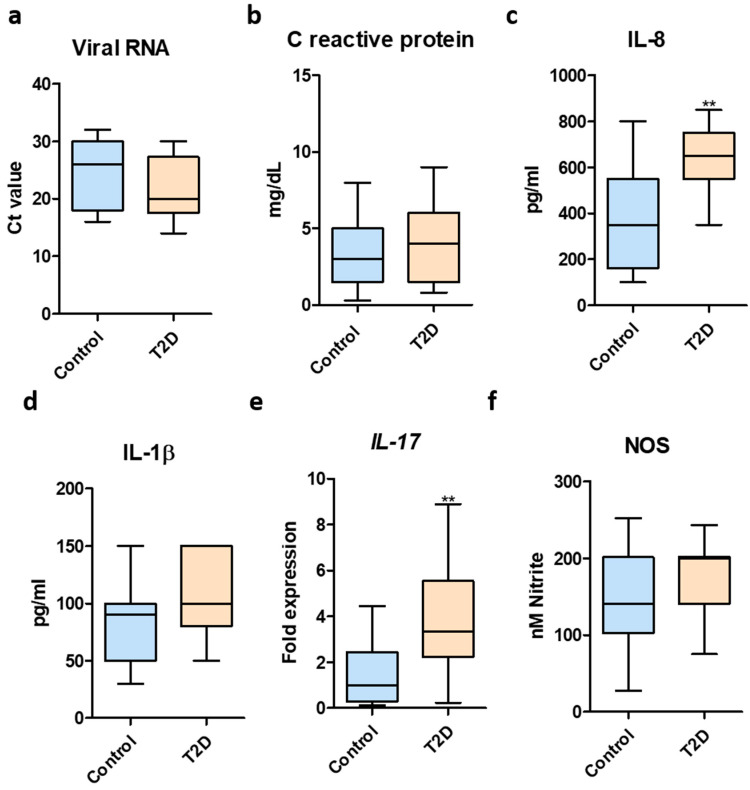
Inflammatory markers in T2D after SARS-CoV-2 infection. (**a**) SARS-CoV-2 RNA levels in healthy controls and T2D patients—determined using RT PCR; (**b**) CRP quantification in serum samples from SARS-CoV-2-infected healthy controls and T2D patients; (**c**) IL-8 quantification in serum samples from SARS-CoV-2-infected healthy controls and T2D patients; (**d**) IL-1β in serum samples from SARS-CoV-2-infected healthy controls and T2D patients; (**e**) IL-17 expression in total blood samples from SARS-CoV-2-infected healthy controls and T2D patients; (**f**) NOS quantification in serum samples from SARS-CoV-2-infected healthy controls and T2D patients. Statistical analyses performed using Unpaired *t*-test with Welch’s correction. **, *p* < 0.01.

Analysis of the SARS-CoV-2 viral RNA levels was performed using Real time PCR in nasopharyngeal swabs. No significant differences were detected between T2D patients and healthy controls (Figure 1a).

The levels of many proinflammatory cytokines (IL-1β, IL-6, IL-8, IL-17, TNF, G-CSF, GM-CSF) and chemokines (IP10, MCP1, MIP1α), were elevated in COVID-19 patients, with higher levels in critically ill subjects [16]. To this end, we examined the levels of proinflammatory markers in the case of SARS-CoV-2-infected T2D subjects compared to infected healthy controls. Even though no significant differences were found in case of CRP levels (Figure 1b), T2D patients exhibited elevated levels of the proinflammatory cytokine IL-8 (Figure 1c). In regards to IL-1β levels, T2D patients had a tendency to harbor higher levels but these were not statistically significant (Figure 1d). Moreover, the expression of the proinflammatory IL-17 gene was significantly higher for the T2D group (Figure 1e). Next, quantification of nitric oxide levels revealed no statistically significant differences between the two tested groups (Figure 1f).

**Figure 2:**
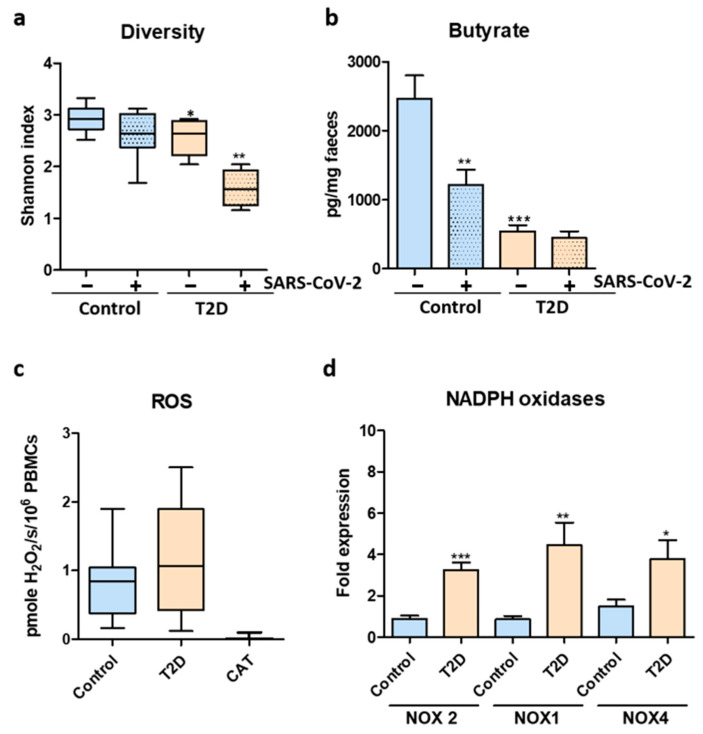
Microbiome dysbiosis and oxidative stress in T2D patients after SARS-CoV-2 infection. (**a**) Microbiome alpha diversity measured by Shannon index. (**b**) Fecal butyrate levels before and after SARS-CoV-2 infection. (**c**) ROS quantification in PBMCs harvested from SARS-CoV-2-infected healthy controls and T2D patients. (**d**) NOX1, NOX2 and NOX4 expression in PBMCs from SARS-CoV-2-infected healthy controls and T2D patient; CT-catalase control. For (**a**,**b**)—statistical analysis was performed by 1-way ANOVA post hoc Bonferroni test and for (**c**,**d**)—statistical analysis was performed using Unpaired *t*-test with Welch’s correction. The statistical significance levels: *, *p* < 0.05; **, *p* < 0.01; ***, *p* < 0.001.

Microbiota analysis was performed using Ion torrent next generation sequencing combined with quantitative Real time PCR. Sequencing of the fecal samples collected from the T2D patients and healthy controls revealed a decreased alpha diversity in the case of T2D samples. As shown by the Shannon index, uninfected T2D patients harbored a decreased diversity of the microbiome (Figure 2a). Quantification of the short chain fatty acid (SCFA) butyrate, an important factor in regulating gut homeostasis, showed that T2D patients had constitutively lower fecal butyrate. Importantly, butyrate levels were significantly altered by SARS-CoV-2 infection in the healthy control group (Figure 2b).

PBMCs isolated from infected healthy controls and T2D patients produced higher levels of hydrogen peroxide, as quantified by Amplex Red (Figure 2c). The elevated ROS production was correlated with significantly higher expression of ROS-producing NADPH oxidases NOX1, NOX2 and NOX 4 in the case of infected T2D patients (Figure 2d).

**Figure 3:**
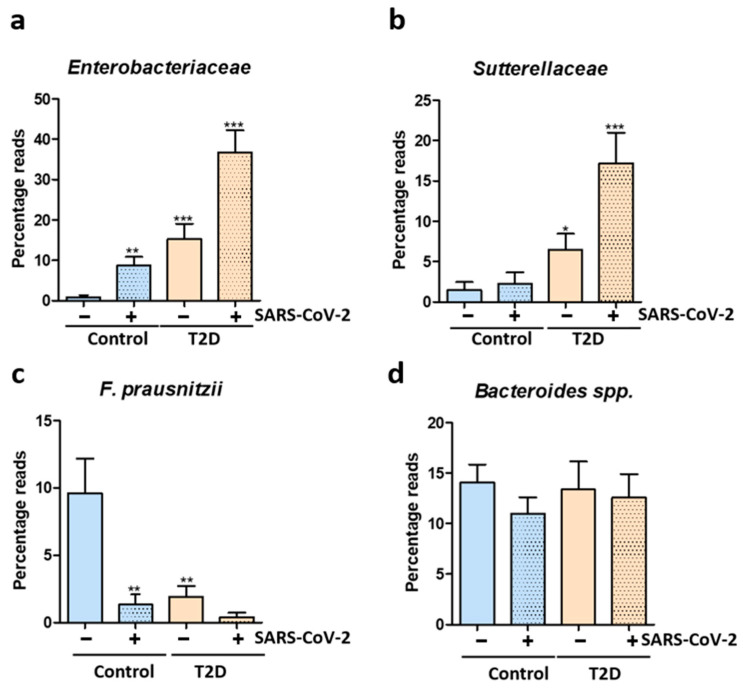
Microbiome alterations after SARS-CoV-2 infection in T2D patients and healthy controls. (**a**) Relative abundance of *Enterobacteriaceae* expressed as percentage reads using next generation sequencing in uninfected and infected T2D patients and healthy controls; (**b**) Relative abundance of *Sutterellaceae* expressed as percentage reads using next generation sequencing in uninfected and infected T2D patients and healthy controls; (**c**) Relative abundance of *F. prausnitzii* expressed as percentage reads using next generation sequencing in uninfected and infected T2D patients and healthy controls; (**d**) Relative abundance of *Bacteroides* spp. expressed as percentage reads using next generation sequencing in uninfected and infected T2D patients and healthy controls. Statistical analysis was performed by 1-way ANOVA post hoc Bonferroni test. The statistical significance levels: *, *p* < 0.05; **, *p* < 0.01; ***, *p* < 0.001.

We next examined into more detail the microbiome changes in healthy controls and T2D patients before and after COVID-19. The main OTUs identified in the analyzed patients included *Bacteroidaceae*, *Enterobacteriaceae*, *Ruminococcaceae*, *Faecalibacterium*, *Lachnospiraceae*, *Rikenellaceae*, *Bifidobacteriaceae*, *Sutterellaceae*, *Clostridiaceae*, *Porphyromonadaceae*, *Desulfovibrionaceae*, *Parasutterella*, *Eubacteriaceae*, *Bilophila*, *Prevotellaceae*, *Alistipes*, *Streptococcaceae*, *Coriobacteriaceae*, *Pasteurellaceae* and *Veillonellaceae*. We found significant alterations of the gut microbiome after SARS-CoV-2 infection in both healthy controls and T2D patients. Indeed, the SARS-CoV-2 infection promoted the blooming of *Enterobacteriaceae* in both tested groups (Figure 3a). In case of T2D patients, we noted constitutively higher *Enterobacteriaceae* (Figure 3a) and *Sutterellaceae* (Figure 3b), which were further enriched after COVID-19.

Conversely, levels of the beneficial microbe *Faecalibacterium prausnitzii* was depleted after SARS-CoV-2 infection, both in healthy controls and in T2D patients (Figure 3c). In case of *Bacteroides* spp., an important and predominant member of the human microbiome, no significant alterations were identified after SARS-CoV-2 infection (Figure 3d).

**Figure 4:**
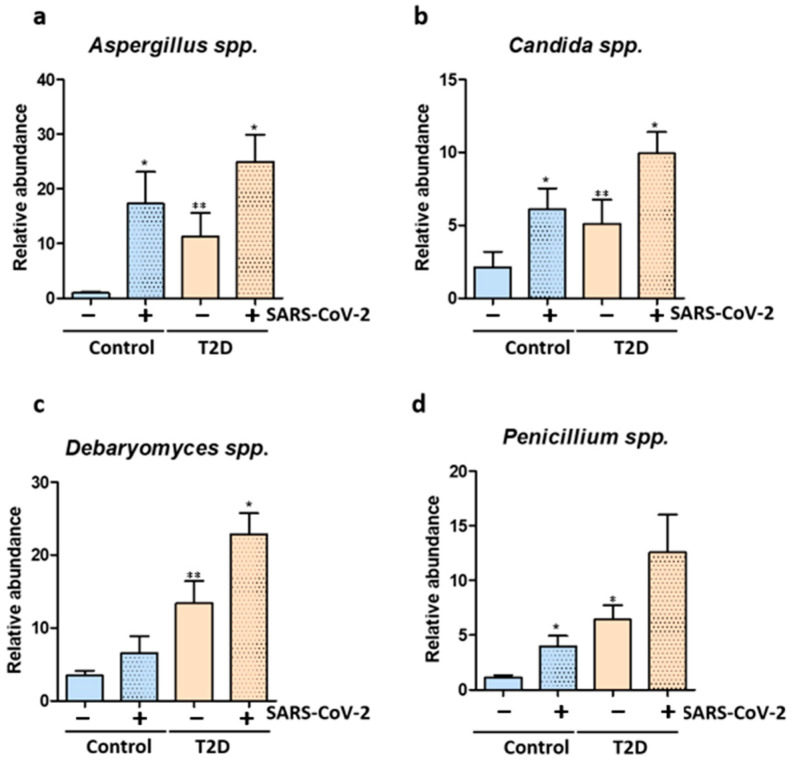
SARS-CoV-2 infection triggers mycobiome changes. (**a**) Relative abundance of *Aspergillus* spp. quantified using Real time PCR in uninfected and infected T2D patients and healthy controls; (**b**) Relative abundance of *Candida* spp. quantified using Real time PCR in uninfected and infected T2D patients and healthy controls; (**c**) Relative abundance of *Debaryomyces* spp. quantified using Real time PCR in uninfected and infected T2D patients and healthy controls; (**d**) Relative abundance of *Penicillium* spp. quantified using Real time PCR in uninfected and infected T2D patients and healthy controls. Statistical analysis was performed by 1-way ANOVA post hoc Bonferroni test. The statistical significance levels: *, *p* < 0.05; **, *p* < 0.01.

We also explored the mycobiome changes before and after SARS-CoV-2 infection. We show that T2D patients, both before and after SARS-CoV-2 infection have a mycobiome enriched in fungi such as *Candida* spp., *Aspergillus* spp., *Debaryomyces* spp. and *Penicillium* spp. (Figure 4a–d). Importantly, SARS-CoV-2 infection induced an enrichment of the *Candida* spp., *Aspergillus* spp., *Debaryomyces* spp. and *Penicillium* spp. fungal populations in the gut of healthy controls (Figure 4a–d).

**Figure 5:**
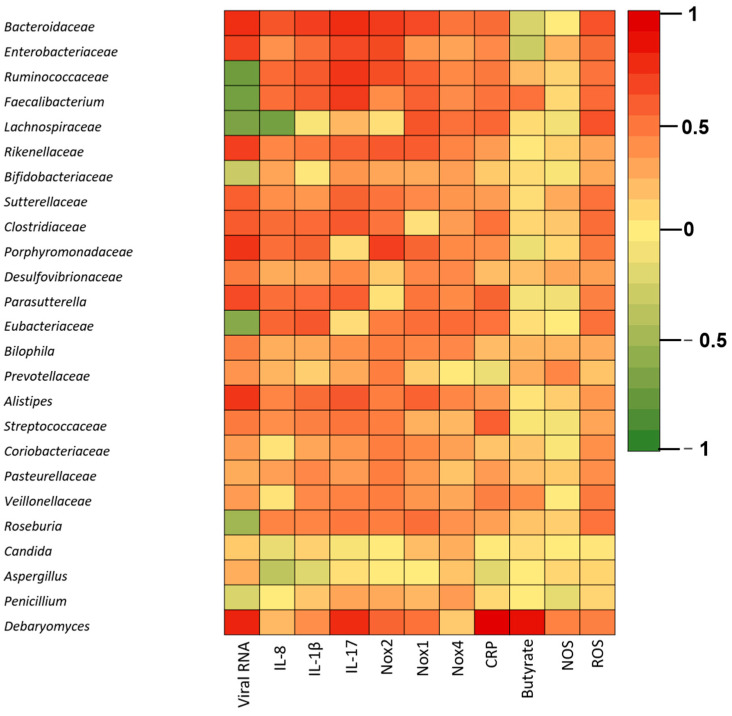
Correlations between clinical parameters and the top OTUs identified in SARS-CoV-2 infected T2D patients and healthy controls. The clinical parameters included viral RNA levels, IL-1β, IL-8, Il-17, oxidative stress (ROS, NOS, Nox1, Nox2, Nox4), CRP. Red indicates a positive correlation, green indicates a negative correlation, while yellow indicates no correlation.

We next correlated the main taxa identified with the patient clinical parameters including viral load, ROS and NOS levels, NADPH oxidase expression, IL-8, IL-1β, IL-17, CRP, and butyrate (Figure 5).

The Spearman correlation analysis revealed significant correlations between the viral load and *Enterobacteriaceae*, *Bacteroidaceae*, *Rickenellaceae* (**** *p* < 0.0001), *Porphyromonadaceae* (**** *p* < 0.0001), *Parasutterella* (**** *p* < 0.0001) and *Alistipes* abundance. Negative correlations between viral RNA levels and the microbiome were identified for *Ruminococcaceae* (**** *p* < 0.0001), *Faecalibacterium* (**** *p* < 0.0001), *Lachnospiraceae* (**** *p* < 0.0001), *Bifidobacteriaceae* and *Eubacteriaceae* (**** *p* < 0.0001).

ROS levels were positively correlated with *Enterobacteriaceae* (** *p* = 0.0019) and *Bacteroidaceae* (*** *p* = 0.0001) and *Faecalibacterium* (** *p* = 0.0017). In exchange, we found no statistically significant correlations between NOS levels and the main microbiota OTUs.

NOX1 expression correlated positively with *Enterobacteriaceae* and negatively with *Lachnospiraceae* and *Faecalibacterium*, Nox2 correlated positively with *Enterobacteriaceae* (**** *p* < 0.0001), Rickenellaceae (*** p = 0.0002), and *Sutterella* (** *p* = 0.0037) and NOX4 correlated positively with *Bacteroidaceae* and negatively with *Lachnospiraceae*.

IL-1β was negatively correlated with Lachnospiraceae (**** p < 0.0001). Conversely, *Enterobacteriaceae* (** *p* = 0.0022), *Parasutterella* (** *p* = 0.0018) and Sutterelaceae (* p = 0.0476) were positively associated with IL-1β. 

IL-8 levels were positively associated with *Enterobacteriaceae* (* *p* = 0.0331), *Sutterella* (* *p* = 0.0289) *Bacteroidaceae* (*** *p* = 0.0002), *Clostridiaceae* (** *p* = 0.0019), and *Parasutterela* (** *p* = 0.0024).

IL-17 expression was positively correlated with *Sutterellaceae* (** *p* = 0.0007), Alistipes (*** p = 0.0002) and *Enterobacteriaceae* (**** *p* ≤ 0.0001). 

We found positive correlations between CRP and several members of the microbiota including *Enterobacteriaceae*, *Alistipes*, *Sutterella* and *Bacteroidaceae*. 

Butyrate levels were negatively correlated with *Bacteroidaceae* and *Enterobacteriaceae* and positively associated with *Faecalibacterium* abundance. 

Further correlations between the mycobiome and the clinical parameters of the T2D patients revealed positive correlations between the viral load and *Debaryomyces* spp. abundance (*p* = 0.0451). Moreover, cytokine levels (IL-1β, IL-8, IL-17) and CRP were positively linked to higher abundance of fungi (*Candida* spp., *Aspergillus* spp. and *Debaryomyces* spp.). Expression of the NADPH oxidase NOX2 was positively correlated with *Debaryomyces* spp.

## 4. Discussion

Dysbiosis has been linked to many immune-related diseases, but still, it remains to be elucidated whether dysbiosis is a cause or consequence of the disease [17]. Similar to other previously published studies, we report the presence of dysbiosis characterized by a dominance of *Enterobacteriaceae* in T2D patients, worsened after COVID-19. These results are consistent with a direct role for gut dysbiosis in enabling dangerous secondary infections and enhancing systemic inflammation during COVID-19.

It is well known that demographic differences can greatly influence the gut microbiome [18]. This is the first study to report changes in the intestinal microbiota triggered by COVID-19 in the Romanian population. The changes identified are similar to those reported in other countries. Studies which originated from different populations in China, USA, Bangladesh, Japan, Portugal, United Arab Emirates, Hungary and Germany reported that the fecal microbiome of patients with COVID-19 has decreased bacterial diversity [19,20,21,22], lower abundance of SCFA-producing bacteria from the *Lachnospiraceae*, *Eubacteriaceae* and *Ruminococcaceae* families as well as an enrichment in opportunistic pathogens from *Enterobacteriaceae* families [20,21,22,23,24,25,26,27,28,29], compared with the fecal microbiome of healthy individuals. The abundance of *Faecalibacterium*, *Lachnospira*, *Eubacterium*, *Roseburia*, *Ruminococcus*, *Coprococcus* was decreased, whereas *Rothia*, *Enterococcus*, *Lactobacillus* levels increased [20,21,22,23,24,25,26,27,28,29,30]. A study from China reported gut dysbiosis in COVID-19 patients with lower levels of probiotic *Lactobacillus* and Bifidobacterium genera [29]. 

*Escherichia coli* and *Klebsiella pneumoniae* are opportunistic pathogens belonging to *Enterobacteriaceae* and were consistently reported to be over-represented in the gut of critically ill COVID-19 patients [19,26]. Expansion of pathobionts is often associated with a disrupted gut barrier leading to a higher risk of bloodstream infections in COVID-19 [31]. In addition, co-infections with *Klebsiella* spp., *Enterococcus* spp. and *E. coli* (species known to include antimicrobial-resistant strains) have been reported in 3–25% of patients in various studies [32,33]. Importantly, up to 50% of deaths in critical patients were caused by these co-infections [34]. Similar to other published reports, we demonstrate here that the abundance of butyrate-producing microbes such as *Faecalibacterium* and *Roseburia* was negatively correlated with disease severity [25,30,35]. A study on the German population (*n* = 117) reported that *Roseburia* and *Faecalibacterium* were negatively associated with disease severity [25]. Another study on 100 patients with COVID-19 and 78 uninfected controls from Hong Kong reported that changes in the composition of the gut microbiota were associated with COVID-19 severity and, similar to our results, with altered levels of inflammatory markers [29]. Loss of bacterial species with potential immunomodulatory activity such as *Eubacterium rectale* and *F. prausnitzii* was correlated with elevated serum levels of proinflammatory mediators, TNF C-X-C motif ligand 10 (CXCL10) and C-X-C motif ligand 2 (CXCL2), but also with increased plasma levels of the anti-inflammatory cytokine IL-10 [29].

We report reduced levels of the SCFA butyrate in the feces of both control and T2D patients. Indeed, several studies have reported low SCFA synthesis in fecal samples of SARS-CoV-2-infected individuals [11]. In a metagenomic study, which analyzed 66 antibiotics-naive COVID-19 patients and 70 uninfected individuals, the infected patients exhibited a reduced capacity of gut microbial SCFA biosynthesis, which was negatively correlated with disease severity [11]. In another study, 19 patients with severe and/or critical SARS-CoV-2 infection were characterized by low fecal concentrations of SCFAs, including butyrate, propionate, acetate, caproic acid and valeric acid [11]. SCFAs can set out the anti-inflammatory responses of immune cells, inhibit inflammatory signaling cascades [36] and maintain the integrity of the intestinal barrier to avoid the translocation of microbes and their endotoxins into the bloodstream [37]. Since SCFAs are important actors in regulating host immune responses, the deficiency in SCFA biosynthesis in SARS-CoV-2 infections could be correlated with disease pathogenesis and severity. Nevertheless, whether SCFA depletion is a cause or consequence of COVID-19 infection remains to be elucidated.

The microbiome is not just home to bacteria but also to a large number of archaea, viruses and fungi, which altogether impact the host physiology and response to infection. So far, only two observational studies demonstrated that COVID-19 was linked to altered composition of fungal microbiota [38,39]. Patients with COVID-19 (*n* = 67) as well as patients with H1N1 Influenza infection (*n* = 35) harbored an increased fungal load with *Aspergillus* and *Penicillium* [39]. Moreover, COVID-19 patients also exhibited a heterogeneous mycobiome with higher loads of opportunistic fungal pathogens, including *Candida albicans*, *Aspergillus flavus* and Candida auris [38]. This could explain the prevalence of *Candida* spp. infections, ranging from 0.7% to 23.5% in COVID-19 patients, considered a major complication in severe cases [40]. Thus, future studies are needed to examine changes in non-bacterial microbiome in COVID-19 patients.

Substantial evidence points towards a greater risk of more severe COVID-19 outcomes in individuals with T2D and obesity, two frequently co-existing conditions [41,42,43]. As a routinely used medication in T2D, metformin has not only a hypoglycemic activity, but also impacts the gut microbiome by enhancing the SCFAs levels, which harbor anti-inflammatory activity [44]. In our study, all participants were on metformin treatment both before and after COVID-19. The fact that none of the patients analyzed within this study had severe SARS-CoV-2 infection sustains the hypothesis that metformin holds an important antiviral role and may protect against severe COVID-19. In line with this, it was reported that metformin activates the protein kinase by AMP (AMPK), leading to the phosphorylation of ACE2. This in turn leads to conformational and functional changes in the surface protein that may cause a decrease in the SARS-CoV-2 binding capacity [45].

Most studies reported so far on the COVID-19-host microbiome interplay have focused on comparing infected and uninfected controls [26,27,28,29,30,46] and, importantly, the majority of the patients analyzed in these studies did not have any co-morbidities [8] or in some cases only a small percentage of the subjects had T2D [9,46]. For instance, Sun et al. recently reported that hypertension and diabetes did not significantly impact the microbiome in a cohort of 63 COVID-19 patients but from the total of patients analyzed only 7 had diabetes [46]. The novelty of our study is the comparison between healthy and T2D patients, before and after COVID-19. In comparison with most studies reported on this subject that are cross-sectional, our study is longitudinal, since the same set of patients has been analyzed before and after COVID-19 infection. Despite the small sample size, we have been able to find some significant changes regarding microbiome patterns after SARS-CoV-2 infection. Clinical information and symptoms at the time of sample collection were not always well presented, so this has hindered the ability to perform more extensive statistical correlations. However, the changes in microbiota could be correlated with inflammatory and oxidative stress markers. 

Consistent gut microbial changes have been demonstrated for COVID-19 patients. Nevertheless, all studies reported so far have some limitations. The effect of viral variants of concern, such as Omicron, on the gut microbiota is still unexplored, as most published studies so far have focused on earlier virus variants. Moreover, most studies have focused on adult populations and the effect of SARS-CoV-2 on the children gut microbiome in children is less understood. 

Last but not least, SARS-CoV-2 infection not only causes damage to the respiratory tract but can affect other organs. Emerging studies have connected COVID-19 with the onset of preeclampsia during pregnancy [47], problems of the reproductive system in men (i.e., deterioration in semen parameters) [48] and neurological complications [49]. Hence, future studies are needed in order to better understand the virus–host–microbiome interplay so that improved therapeutic approaches can be developed.

## 5. Conclusions

Emerging preclinical and clinical studies suggest that the gut microbiome might impact COVID-19 pathogenesis and outcome. It is paramount to enrich our knowledge on the impact of microbiota diversity and associated immunological mechanisms on SARS-CoV-2 severity and take it into account when modelling COVID-19 infection–fatality ratios. 

Next, we need mechanistic studies to investigate the impact of specific microbial communities (bacteria as well as fungi) and viruses on COVID-19 pathogenesis and clinical outcomes.

## Figures and Tables

**Table 1:** Patient characteristics: age, sex (female/male), body mass index (BMI), blood pressure (millimeters of mercury—mmHg), glycated hemoglobin (HbA1c), high-density lipoprotein (HDL), low-density lipoprotein (LDL), tryglycerides (TG), and medication used—statin, metformin, Dipeptidyl peptidase-4 (DPP4) inhibitors and insulin.

Characteristic	HC	T2D
Age	56 ± 10.30	63 ± 12.25
Sex (F/M)	10/5	9/6
BMI	24.8 ± 2.25	31 ± 4.29
Blood pressure (mmHg): systolic	110 ± 2.10	138.5 ± 2.8
Blood pressure (mmHg): diastolic	62 ± 1.99	88.1 ± 1.3
HbA1c (%)	5.4 ± 0.19	6.5 ± 0.6
HDL (mg/dL)	65 ± 3.99	47 ± 6.55
LDL (mg/dL)	97 ± 15.56	118 ± 27.67
TG (mg/dL)	88 ± 14.27	132 ± 48.47
Statin (number/total)	2/15	3/15
Metformin (number/total)	n/a	15/15
DPP4 inhibitors	n/a	2/15
Insulin	n/a	0/15

**Table 2:** Sequences of primers used.

Target	Sequence
*Penicillium* spp.	ATTGGAGGGCAAGTCTGGTG
	AATCCCGTCCGATCCCTAGT
RNAr 18S	ATTGGAGGGCAAGTCTGGTG
	CCGATCCCTAGTCGGCATAG
*Debaryomyces* spp.	TAACGGGAACAATGGAGGGC
	CAACACCCGATCCCTAGTCG
*Candida* spp.	TTTATCAACTTGTCACACCAGA
	ATCCCGCCTTACCACTACCG
*Aspergillus* spp.	GTGGAGTGATTTGTCTGCTTAATTG
	TCTAAGGGCATCACAGACCTGTT

## Data Availability

The data presented in this study are available on request from the corresponding author. The data are not publicly available due to privacy/ethical restrictions.
